# Chemistry of polyhalogenated nitrobutadienes, 10: Synthesis of highly functionalized heterocycles with a rigid 6-amino-3-azabicyclo[3.1.0]hexane moiety

**DOI:** 10.3762/bjoc.8.69

**Published:** 2012-04-23

**Authors:** Viktor A Zapol’skii, Jan C Namyslo, Armin de Meijere, Dieter E Kaufmann

**Affiliations:** 1 Institute of Organic Chemistry, Clausthal University of Technology, Leibnizstr. 6, 38678 Clausthal-Zellerfeld, Germany; 2 Institute of Organic and Bioorganic Chemistry, Georg-August-University Göttingen, Tammannstrasse 2, 37077 Göttingen, Germany

**Keywords:** isothiazole, nitrochlorobutadiene, nitropyrazole, nucleophilic substitution, pyrimidine, small rings

## Abstract

The nitropolychlorobutadienes **3**, **4** are valuable building blocks for various amination and successive heterocyclization products. Nucleophilic substitution reactions of the partially protected, bioactive amines **1**, **2** with either vinyl, imidoyl or carbonyl chlorides result in the formation of the enamines **11**, **12**, **13**, **16**, **25**, the amidine **6**, and the amides **20**, **21**, respectively. In the following, cyclization to the highly functionalized pyrazoles **27**, **28**, pyrimidine **26** and pyridopyrimidine **24** succeeded. Deprotection of **21**, **12** and **28** proved to be only partially feasible.

## Introduction

Nitropolychlorobutadienes are potent precursors for a variety of highly functionalized acyclic and (hetero)cyclic compounds. The readily accessible 2-nitroperchloro-1,3-butadiene (**3**) [1] is one of the most prominent members of this rather new class of dienes. During the past nine years we have published the syntheses of a wide range of diverse substance classes, applying this useful starting material [2–7].

**Figure 1:**
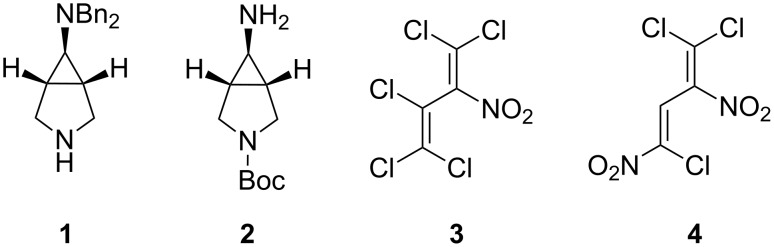
Promising starting materials for biologically active compounds.

The present work focuses on pharmacologically promising derivatives of the protected 6-amino-3-azabicyclo[3.1.0]hexanes **1** and **2**, which are obtained upon reaction with polychloronitrobutadienes **3** and **4** (Figure 1).

**Figure 2:**
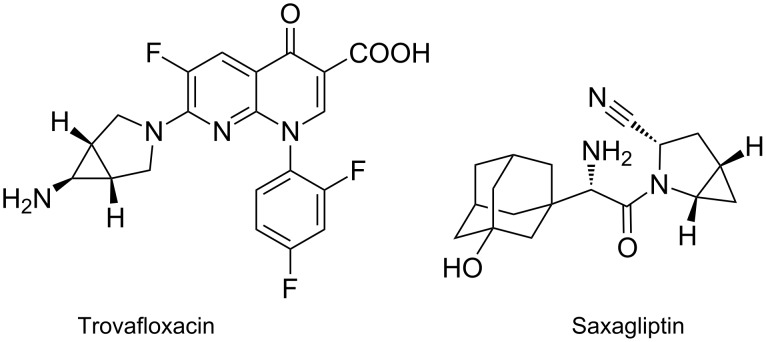
Pharmaceuticals bearing an azabicyclo[3.1.0]hexane unit.

The rigid bisamine 6-amino-3-azabicyclo[3.1.0]hexane is an essential building block of several pharmaceuticals, such as the potent gyrase inhibitor Trovafloxacin (Figure 2) [8–9]. As a 4^th^ generation topoisomerase inhibitor, this fluoroquinolone anticipates replication of the bacterial DNA [10]. Other azabicyclo[3.1.0]hexane derivatives, for example with oxo-, oxazolidino-, quinolino-, oxobenzothiazolo[3,2-*a*]quinolino, or pyrrolidino substituents, exhibit remarkable antibacterial as well as antiprotozoal activity [11–15]. Furthermore, the 2-azabicyclo[3.1.0]hexane derivative of 3-hydroxyadamantylglycine, named Saxagliptin, is a pharmaceutical of the dipeptidyl peptidase IV (DPP-4) inhibitor class against type 2 diabetes mellitus and entered the market in 2009 (Figure 2) [16].

## Results and Discussion

**Scheme 1:**
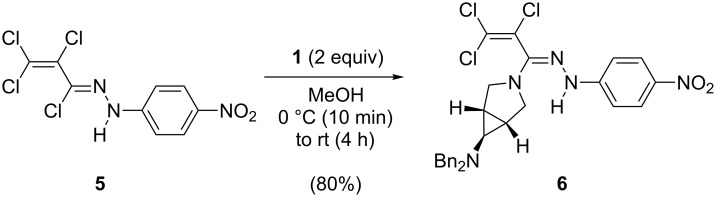
Synthesis of the azabicyclic hydrazone **6**.

Driven by the promising stepwise reactivity of the highly substituted butadienes **3**, **4** and with the above mentioned hints to biological activities in mind, we set out to develop structural conjunctions of the nitropolychlorobutadienes **3** and **4** with the 3-azabicyclo[3.1.0]hexane building blocks **1** and **2**. Upon treatment with *N*,*N*-dibenzyl-3-azabicyclo[3.1.0]hexan-6-amine (**1**) in methanol the (tetrachloroallylidene)hydrazine **5** [4,7] derived from **3** reacted in a formal nucleophilic substitution at the imidoyl chloride unit to give the derivative **6** in 80% isolated yield (Scheme 1). The interest in such compounds derives from the fact that a number of similar hydrazones, e.g., phenyl(phenylchloromethylidene)hydrazine, exhibit fungicidal, antibacterial, and fungistatic activity [17–18].

**Scheme 2:**
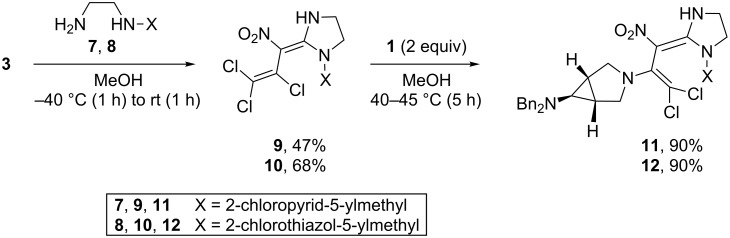
Novel imidacloprid analogues **11**, **12**.

In view of the insecticidal properties of some recently published [19] analogues of imidacloprid (*N*-[1-[(6-chloro-3-pyridyl)methyl]-4,5-dihydroimidazol-2-yl]nitramide), the substitutions of the imidazolidines **9** and **10** with **1** were also tested. Compounds **9** and **10** were prepared from the nitrodiene **3** and the ethylenediamines **7** and **8**. The formal nucleophilic substitution of the α-chloro substituent within the trichlorovinyl group of **9** and **10** by the amine **1** proceeded smoothly to give both of the novel imidacloprid analogues **11** and **12**, each in 90% yield (Scheme 2).

**Figure 3:**
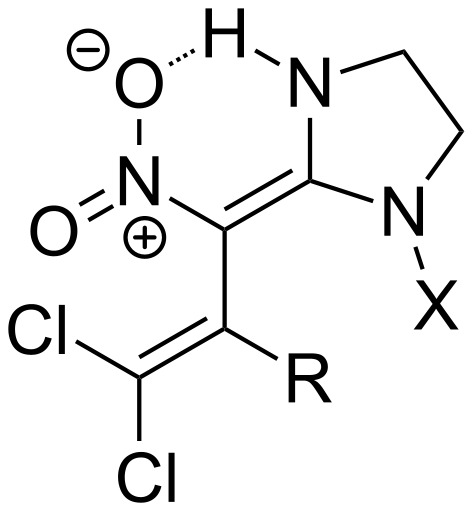
Stabilizing hydrogen bond in nitrobutadiene-derived imidacloprid analogues **9**–**12**.

It is worth noting that all these nitroenamines **9**–**12** are formed as *E*-isomers, which are stabilized by an intramolecular hydrogen bridge bond in a six-membered pseudocycle (Figure 3).

**Scheme 3:**
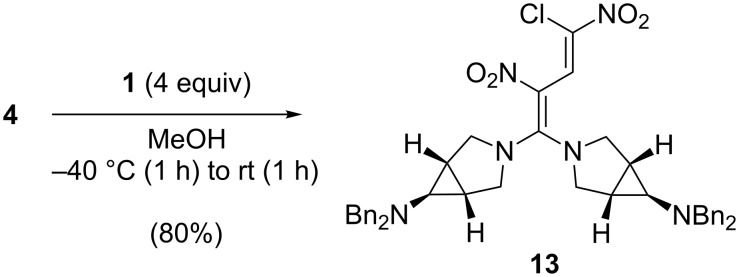
Synthesis of the 4,4-bis(aminoazabicyclo[3.1.0]hexyl)-1-chloro-1,3-dinitrobutadiene **13**.

Upon treatment of 1,3-dinitro-1,4,4-trichlorobutadiene (**4**), which was obtained in a four-step sequence from 1,2-dichloroethylene (mixture of diastereomers) [20–21], with a fourfold excess of the azabicyclo[3.1.0]hexane **1** in methanol at −40 °C, a twofold vinylic substitution led to the 4,4-bis(aminoazabicyclo[3.1.0]hexyl)-1-chloro-1,3-dinitrobutadiene **13** in 80% yield (Scheme 3).

**Scheme 4:**
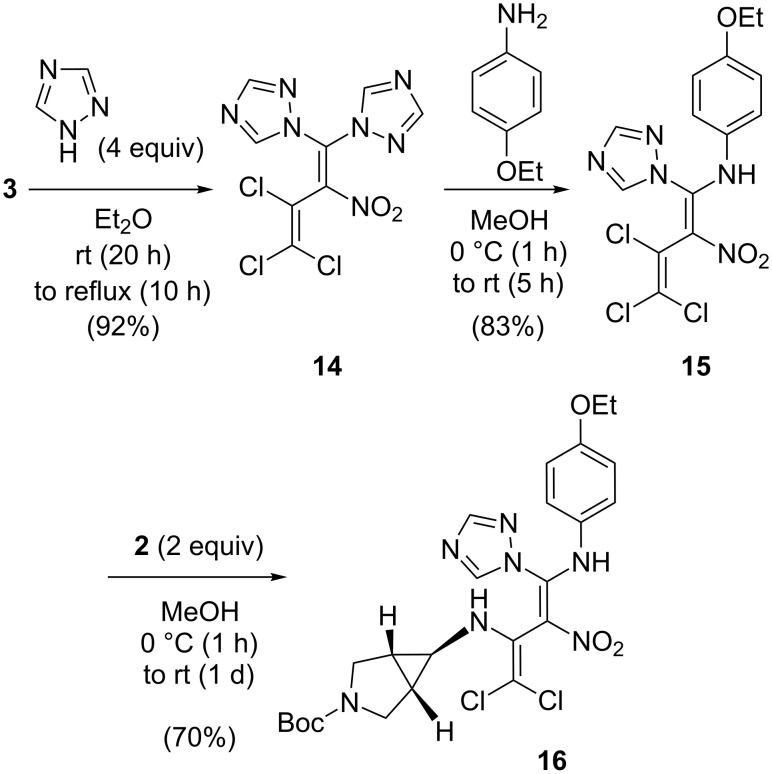
Synthesis of the highly substituted trisaminodichloronitrobutadiene **16**.

In an analogous treatment of the pentachloronitrobutadiene **3** with a fourfold excess of 1,2,4-triazole in diethyl ether the 4,4-bistriazolyltrichloronitrobutadiene **14** (92% yield) was obtained [22] and turned out to be an appropriate substrate for a transamination, as the triazole is an excellent leaving group. Thus, by treatment of **14** with *p*-phenetidine the 4-triazolyl-4-(4-ethoxyphenylamino)butadiene **15** was obtained in 83% yield. Subsequent reaction of **15** with *tert*-butyl 6-amino-3-azabicyclo[3.1.0]hexane-3-carboxylate (**2**) provided the tris(amino)butadiene **16** in 70% yield (Scheme 4).

**Figure 4:**
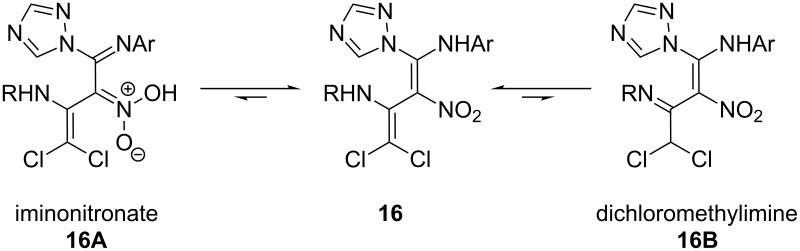
Conceivable tautomeric structures of **16**.

It is noteworthy that the diaminonitrovinyl moiety in compound **15** remains unaffected in the last substitution step. Apparently, the 2-nitroenamine substructure in **15** is less electrophilic at C-3 than its conceivable tautomeric structure **16A** would suggest. After the third formal nucleophilic substitution at C-2, the resulting product **16** has a second enamine substructure as in **16A**, rather than a dichloromethylimine subunit as in **16B**. This is obvious from the ^1^H NMR spectrum of **16**, which does not show a signal corresponding to a dichloromethyl proton, and in addition the ^13^C NMR spectrum shows four signals associated with olefinic carbon atoms (Figure 4).

**Scheme 5:**
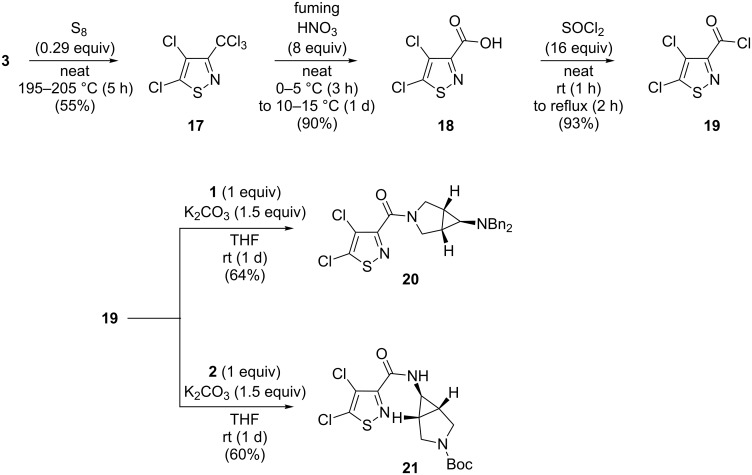
Syntheses of the perfunctionalized isothiazole derivatives **20**, **21**.

In addition to the direct attachment of heteroatoms or even heterocycles to nitropolychlorobutadienes by formal vinylic nucleophilic substitution reactions as described above, it was of interest to incorporate a persubstituted diene unit as in **3** and **4** into a heterocycle. For example, the isothiazole **17** was obtained from the nitrodiene **3** upon treatment with elemental sulfur at 200 °C [23]. Subsequent reaction with fuming nitric acid provided the dichloroisothiazolocarboxylic acid **18** [24], which could be easily converted with thionyl chloride to the corresponding acid chloride **19** (93% yield). The latter smoothly reacted with the azabicyclohexane derivatives **1** and **2** to provide the corresponding amides **20** and **21**, respectively (Scheme 5). These amides **20** and **21** are hot candidates for biological testing, as some known amides of 4-chloroisothiazol-3-carboxylic acid have been shown to exhibit high antibacterial activity [25–27].

The high number of heteroatoms in **20** and **21**, accompanied by only a few hydrogen atoms, requires ^1^H/^13^C-2D as well as nitrogen NMR spectra for structural assignments. For example, aside from aromatic protons in **20**, the methylene groups within the pyrrolidine ring each appear as a set of one single doublet (geminal coupling only) and a doublet of doublets (with additional coupling to the bridgehead proton due to an appropriate dihedral angle). Narrow shifts of the corresponding carbon atoms were assigned by means of an HSQC spectrum. The benzylic methylene protons give two slightly separated doublets (each with the expected ^2^*J* coupling of about 13 Hz). Appearing at 3.59 and 3.54 ppm, respectively, they are attached to isochronous carbon atoms at 59.0 ppm. Furthermore, the proton of the NCH fragment (^13^C NMR: δ = 47.3 ppm) of the cyclopropane ring appears as a triplet at 1.54 ppm (*J* = 2.3 Hz). Interestingly, the chemical shift of both of the bridgehead protons is 1.35 ppm (dd, *J* = 4.2, 2.3 Hz), whereas the corresponding carbon atoms have slightly different chemical shifts of 25.5 and 24.5 ppm, respectively. Most of the quarternary carbon shifts are unambiguous, whereas an HMBC spectrum was necessary for the assignment of two downfield signals: 160.5 ppm (C=O) and 160.4 ppm (SC). ^14^N NMR (one-dimensional, direct detection) and an inverse-detected ^1^H/^15^N-HMBC gave the nitrogen shifts (internal MeNO_2_ at 0.0 ppm): −65.8 ppm (C=N), −244.2 ppm (NCO), and −319.5 ppm (NBn).

**Scheme 6:**
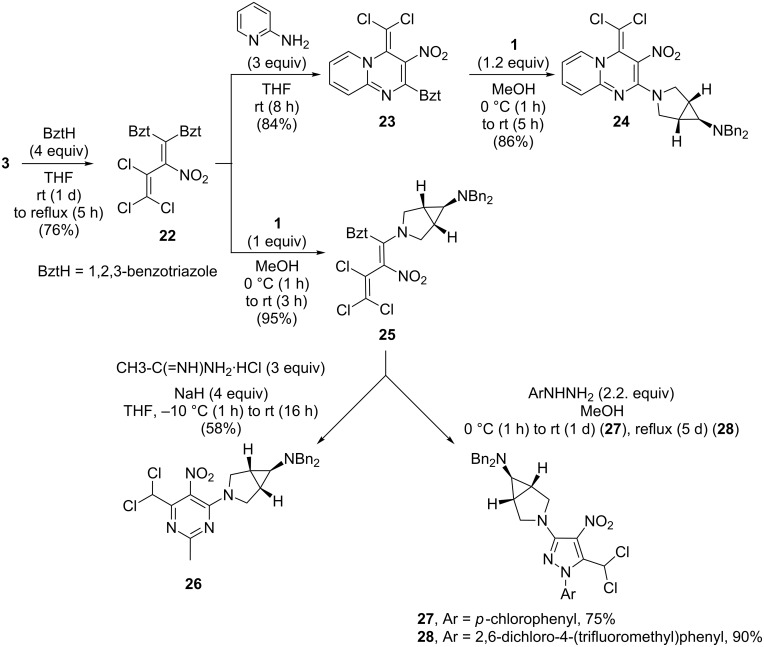
Preparation of the pyrazoles **27**, **28**, the pyrimidine **26** and the pyridopyrimidine **24**.

In addition to the twofold triazole substitution, the nitrodiene **3** was treated with four equiv of benzotriazole in THF. Thus, the bisbenzotriazole derivative **22** was obtained in 76% yield [28]. Having the target of further compounds with insecticidal activities in mind, **22** was treated with 2-aminopyridine, but the simple substitution product, a benzotriazolyl-1-(pyrid-2-ylamino)diene, which must have been formed initially, apparently must have tautomerized to a pyridin-2(1*H*)-imine derivative, which then underwent cyclization by a formal nucleophilic substitution leading to the 4*H*-pyrido[1,2-*a*]pyrimidine **23**. The remaining benzotriazole group in **23**, which is activated by the adjacent nitro substituent allows for a further nucleophilic substitution. Therefore, upon treatment with the azabicyclohexane **1** under mild conditions (methanol, rt) the enamine **24** was formed in 86% yield (Scheme 6). Similar pyrido[1,2-*a*]pyrimidines show antiviral [29], antithrombotic [30] and antibacterial [31–34] activities.

Alternatively, conversion of bis(benzotriazolyl)butadiene **22** with one equiv of amine **1** led to transamination at C-1 of the butadiene to furnish the azabicyclohexyl-nitrobutadiene **25** in 95% yield. The latter, on one hand was converted by treatment with acetamidine hydrochloride in THF to the pyrimidine **26** (yield 58%), which apparently proceeds by transamination and subsequent intramolecular S_N_Vin reaction. The structure of an analogous derivative of this heterocycle was previously confirmed by X-ray crystallography [5]. On the other hand, nitrodiene **25** was treated with arylhydrazines to give the persubstituted aminonitropyrazoles **27**–**28** in 75–90% yield (Scheme 6).

**Scheme 7:**
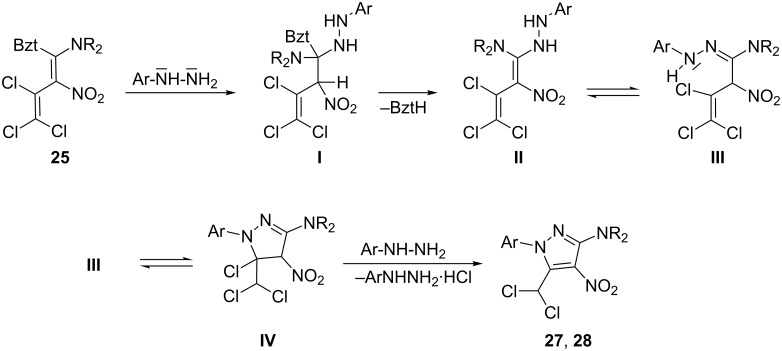
Proposed reaction mechanism for the formation of **27**, **28**.

A conceivable mechanism for the cascade reaction that leads to the pyrazoles **27** and **28** is presented in Scheme 7. Initially, the trichlorobutene **I** is formed upon addition of the arylhydrazine to the nitro-substituted butadiene **25**. Subsequent elimination of benzotriazole results in the diaminobutadiene **II**, which tautomerizes to the stable amidine **III**. The pyrazoline **IV** is then formed by an intramolecular S_N_Vin reaction. Finally, HCl elimination affords the pyrazoles **27**, **28**. The stimulus to investigate such compounds originated from the known pharmacological activities of 4-nitropyrazoles [35–42].

**Scheme 8:**
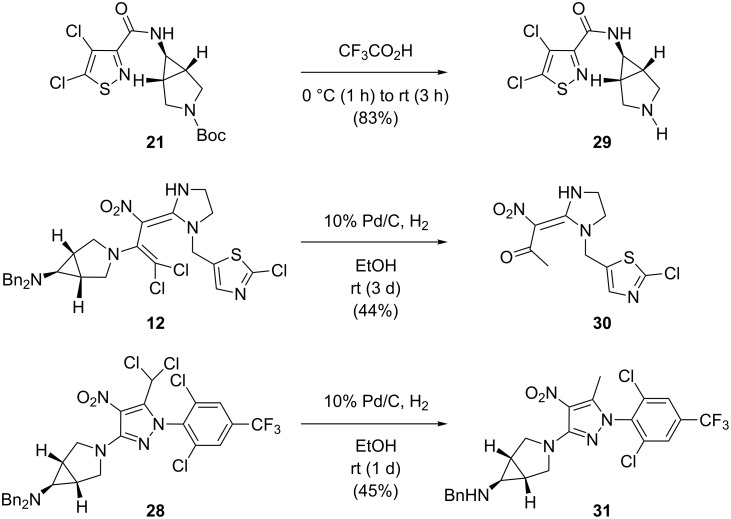
Attempted deprotection of the azabicyclic compounds **21**,**12**, and **28**.

At the end of our synthetic work, three of the intricate 6-amino-3-azabicyclo[3.1.0]hexane derivatives were subjected to common deprotection conditions on a micromole scale (Scheme 8). Interestingly, in the case of the removal of the *N*-Boc group from **21** by means of surplus trifluoroacetic acid under mild conditions, the corresponding free amine **29** was obtained in 83% yield, without any further optimization. However, accurately tailored conditions seemed to be necessary for the N-debenzylation of the protected amino compounds **12** and **28**. The application of the usual reductive conditions (i.e., hydrogen under atmospheric pressure, palladium on charcoal suspended in, e.g., ethanol) to the protected amine **12** led to a multiple reaction involving the deprotection, a bisdechlorination and final hydrolysis of an intermediate imine. The resulting ketone **30** was isolated in 44% yield. On the other hand, the highly substituted dibenzylamino compound **28** showed another interesting reaction pathway: One of its benzyl groups was unmodified, even though the competing reduction of the dichloromethyl substituent took place to give the mono-N-benzylated rigid amine **31** in 45% yield. With Pd/C at ambient hydrogen pressure and ethanol as a solvent, no reduction of the nitro group or the aromatic chlorine atoms in **12** and **28** was observed. To avoid the described side reactions, further experiments should comprise the optimization of the hydrogen volume and pressure as well as some fine tuning of the catalyst/solvent system.

To the best of our knowledge, the transformation observed for compound **12**, i.e., the reduction of a 1-amino-2,2-dichlorovinyl group to an acetyl substituent, is hitherto unprecedented. However, the individual parts of these multistep reactions, namely the conversion of the 1-amino-2,2-dichlorovinyl group to a dichloromethyl ketone and, in addition, the reductive bisdechlorination of a dichloromethyl group were recently published by our group [5,43].

## Conclusion

Regioselective reactions of the nitrotrichlorobuta-1,3-dienes **3** and **4**, some after initial transformations to other derivatives with *exo*-6-*N*,*N*-dibenzylamino-3-azabicyclo-[3.1.0]hexane (**1**) and *exo*-6-amino-3-(*tert*-butoxycarbonylaza)bicyclo[3.1.0]hexane (**2**), led to a series of potentially biologically active compounds, which are due to be tested in various assays.

## Supporting Information

File 1 Experimental section.
