# Supplementary material for: Chemistry of polyhalogenated nitrobutadienes, 10: Synthesis of highly functionalized heterocycles with a rigid 6-amino-3-azabicyclo[3.1.0]hexane moiety
Source: Beilstein J Org Chem. 2012 Apr 23;8:621–8. doi: 10.3762/bjoc.8.69 (PMC3343288; doi:10.3762/bjoc.8.69)
Supplement: File 1 — Experimental section. [file Beilstein_J_Org_Chem-08-621-s001.pdf]

Supporting Information

for

Chemistry of polyhalogenated nitrobutadienes, 10: Synthesis of highly functionalized heterocycles with a rigid 6-amino-3-azabicyclo[3.1.0]hexane moiety

Viktor A. Zapol'skii[1], Jan C. Namyslo[1], Armin de Meijere[2] and Dieter E. Kaufmann*[1]

Address: [1]Institute of Organic Chemistry, Clausthal University of Technology, Leibnizstr. 6, 38678 Clausthal-Zellerfeld, Germany and [2]Institute of Organic and Bioorganic Chemistry, Georg-August-University Göttingen, Tammannstrasse 2, 37077 Göttingen, Germany

Email: Dieter E. Kaufmann* - dieter.kaufmann@tu-clausthal.de

*Corresponding author

Experimental section

General

Melting points were determined with a Büchi apparatus 520 and are uncorrected. ^{1}H NMR, ^{1}H-decoupled ^{13}C NMR, and ^{15}N NMR spectra were recorded with a Bruker Avance 400 instrument from solutions in $CDCl_3$, acetone-d_6, or DMSO-d_6. All NMR data are reported downfield from TMS as the internal standard, unless otherwise stated. IR spectral data were obtained for solids as KBr discs on a Bruker Vector 22 FT-IR. Mass spectra were obtained

on a Hewlett Packard MS 5989B spectrometer, usually in direct mode with electron impact ionization (70 eV). In the case of chlorinated compounds, all peak values of molecular ions as well as fragments refer to the isotope ^{35}Cl. The elemental composition was confirmed by high-resolution ESI, EI, or CI mass spectrometry. The ESI measurements were done on a ESI-TOF-HR-MS MicroTOF (Bruker Daltonik) at the Institute of Organic and Biomolecular Chemistry, University of Göttingen, Germany, or on a Waters Micromass LCT or Q-TOF Premier at the Institute of Organic Chemistry, Leibniz University, Hannover, Germany. At the same site the HR-EI mass spectrometry was done on a Micromass VG Autospec (70 eV). All HRMS results were satisfactory in comparison with the calculated accurate mass of the molecular ion (+/−2 ppm, R ~10000); for this reason, only calculated values are stated. TLC was performed on Merck TLC plates (aluminium based) silica gel 60 F 254. Column chromatography was carried out on silica gel 60 (Merck). MeOH was purchased as reagent grade and used as received. Petroleum ether had a boiling range of 60–70 °C.

Exo-6-*N*,*N*-dibenzylamino-3-azabicyclo[3.1.0]hexane (**1**) and *exo*-6-amino-3-(*tert*-butoxycarbonylaza)bicyclo[3.1.0]hexane (**2**) were synthesized according to published protocols [1] from *N*-Boc-δ^3-pyrroline. 2-Nitropentachlorobuta-1,3-diene (**3**) was synthesized according to the literature [2] from 2*H*-pentachlorobuta-1,3-diene with a 10:1 solution of 63% HNO_3 and 98% H_2SO_4 in 53% yield (bp 69–71 °C, 1 mbar). 1,3-Dinitro-1,4,4-trichlorobuta-1,3-diene (**4**) was synthesized according to the published procedure [3].

N-(1-(6-Dibenzylamino-3-azabicyclo[3.1.0]hex-3-yl)-2,3,3-trichloro-allylidene)-*N*'-(4-nitro-phenyl)hydrazine (**6**)

The hydrazone **5** was prepared from the nitrodiene **3** and *p*-nitroaniline according to the published procedure [4]. To a suspension of the hydrazone **5** (500 mg, 1.52 mmol) in MeOH (20 mL) was added a solution of the amine **1** (890 mg, 3.19 mmol) in MeOH (5 mL) with stirring at 0 °C, within 10 min. The resulting reaction mixture was stirred for 4 h at rt. Subsequently, the supernatant liquid was concentrated in vacuo to a volume of about 10 mL and treated with cold HCl (5%, 30 mL). The precipitate was isolated, washed with H_2O (3 ×

50 mL), cold MeOH (1 × 5 mL), and finally dried under reduced pressure. Yield: 692 mg (80%); mp 146–148 °C; IR (KBr) $\tilde{\nu}$: 3342, 2853, 1597, 1579 (NO_2), 1496, 1473, 1314 (NO_2), 1302, 1272, 1212, 1175, 1110, 949, 898, 837, 751, 700 cm^{-1}; ^1H NMR ($CDCl_3$) δ 8.11 (d, J = 9.2 Hz, 2 H, H_{aryl}-3,5), 7.54–7.28 (m, 10 H, Ph), 6.94 (br s, 1 H, NH), 6.88 (d, J = 9.2 Hz, 2 H, H_{aryl}-2,6), 3.68 (br s, 4 H, CH_2Ph), 3.50–3.22 (m, 4 H, NCH_2), 1.66 (br s, 1 H, NCH), 1.60 (br s, 2 H, NCHC*H*); ^{13}C NMR ($CDCl_3$) δ 150.8 (CH), 126.2 (2 CH, C_{aryl}-3,5), 125.5 (CCl), 118.6 (CCl_2), 111.0 (2 CH, C_{aryl}-2,6), 58.9 (2 C, $C_{quat.}CH_2$), 49.1 (NCH_2), 48.7 (NCH_2), 47.2 (NCH), 25.3 (CH), 25.1 (CH); MS: *m*/*z* (%) = 569 [M^+] (2), 534 $[M–Cl]^+$ (3), 432 $[M–NO_2PhNH]^+$ (2), 373 $[M–NBn_2]^+$ (2), 91 (100); HRMS–EI: *m*/*z* calcd for $C_{28}H_{27}Cl_3N_5O_2$ $[M+H]^+$: 570.1230.

The imidazolidines **9, 10** were synthesized from the nitrodiene **3** and *N*[1]-((6-chloropyridin-3-yl)methyl)ethane-1,2-diamine (**7**) and *N*[1]-((2-chlorothiazol-5-yl)methyl)ethane-1,2-diamine (**8**), respectively, according to the literature procedure [5].

N,*N*-Dibenzyl-3-((*E*)-1,1-dichloro-3-(1-((6-chloropyridin-3-yl)methyl)imidazolidin-2-ylidene)-3-nitroprop-1-en-2-yl)-3-azabicyclo[3.1.0]hexan-6-amine (**11**)

A solution of 384 mg (1.0 mmol) of the imidazolidine **9** and 612 mg (2.2 mmol) of the amine **1** in 10 mL of methanol was stirred at 40–45 °C for 5 h. After cooling down to 5 °C, the mixture was diluted with 50 mL of cold water (0 °C) and neutralized by dropwise addition of concentrated hydrochloric acid. The obtained solid was filtered off, washed with water (3 × 20 mL) and dried in vacuo. Yield: 563 mg (90%); mp 146–148 °C; IR (KBr) $\tilde{\nu}$: 3303, 3027, 2915, 2858, 1562 (NO_2), 1516, 1461, 1320 (NO_2), 1299, 1202, 1127, 1105, 1026, 950, 927, 823, 751, 700 cm^{-1}; ^1H NMR ($CDCl_3$) δ 9.54 (br s, 1H, NH), 8.29 (d, J = 2.4 Hz, 1H, $N_{pyr}CH$), 7.60 (dd, J = 8.3, 2.4 Hz, 1H, $C_{pyr}H$), 7.38 (d, J = 8.3 Hz, 1H, $C_{pyr}H$), 7.37–7.20 (m, 10 H, Ph), 4.69 (d, 1 H, 15.5 Hz, $C_{quat.,pyr}CH_2$), 4.16 (d, 1 H, 15.5 Hz, $C_{quat.pyr}CH_2$), 3.82–3.70 (m, 2 H), 3.66–3.56 (m, 4 H), 3.50 (d, 2 H, 9.6 Hz), 3.41 (d, 2 H, 9.6 Hz), 3.18–3.12 (m, 1 H), 3.01 (d, 1 H, 9.6 Hz), 1.76 (br s, 1 H, NCH), 1.32 (br s, 2 H, NCHC*H*); ^{13}C NMR ($CDCl_3$) δ 160.0

(NCN), 151.6 (NCCl), 148.7 ($C_{pyr}H$), 138.8 ($C_{quat.}$), 138.0 ($C_{pyr}H$), 136.3 ($C_{quat.}$), 129.7 ($C_{q(pyr)}$), 129.4 (4 CH), 128.0 (4 CH), 126.9 (2 CH), 124.7 ($C_{Pyr}H$), 106.0 (=CCl_2), 100.6 (CNO_2), 59.0 (2 C, $C_{quat.,phenyl}CH_2$), 53.0 (CH_2), 51.6 (CH_2), 49.7 (CH_2), 48.7 (CH_2), 47.2 (NCH), 41.7 ($NHCH_2$), 25.9 (CH), 25.5 (CH); MS: *m*/*z* (%) = 624 [M^+] (3), 498 [M–Cl-pyridyl-CH_2]$^+$ (2), 428 [M–NBn_2]$^+$ (2), 249 (100); HRMS–EI: *m*/*z* calcd for $C_{31}H_{32}Cl_3N_6O_2$ [M+H]$^+$: 625.1652.

N,*N*-Dibenzyl-3-((*E*)-1,1-dichloro-3-(1-((2-chlorothiazol-5-yl)methyl)imidazolidin-2-ylidene)-3-nitroprop-1-en-2-yl)-3-azabicyclo[3.1.0]hexan-6-amine (**12**)

Compound **12** was synthesized from 1.0 mmol of the imidazolidine **10** and the amine **1** according to the procedure employed for compound **11**. Yield: 568 mg (90%); mp 133–135 °C; IR (KBr) $\tilde{\nu}$: 3311, 3027, 2917, 2857, 1564 (NO_2), 1510, 1422, 1324 (NO_2), 1303, 1202, 1126, 1044, 937, 751, 700 cm^{-1}; ^1H NMR ($CDCl_3$) δ 9.52 (br s, 1H, NH), 7.47 (s, 1H, $N_{thiazolyl}CH$), 7.38–7.14 (m, 10 H, Ph), 4.75 (dd, 1 H, 0.8, 15.5 Hz, $C_{thiazolyl}CH_2$), 4.36 (dd, 1 H, 0.8, 15.5 Hz, $C_{thiazolyl}CH_2$), 3.83–3.37 (m, 10 H), 3.18 (dd, J = 9.6, 3.6 Hz, 1 H), 3.02 (d, J = 9.6 Hz, 1 H), 1.79 (br s, 1 H, NCH), 1.34–1.30 (m, 2 H, NCHC*H*); ^{13}C NMR ($CDCl_3$) δ 159.1 (NCN), 153.6 (NCCl), 140.8 ($C_{thiazolyl}H$), 138.8 ($C_{quat.,phenyl}$), 136.2 (N=$C_{quat.}$), 134.9 ($C_{quat.,thiazolyl}$), 129.4 (4 CH), 128.0 (4 CH), 126.9 (2 CH), 106.0 (=CCl_2), 101.1 (CNO_2), 59.0 (2 C, $C_{quat.,phenyl}CH_2$), 53.0 (CH_2), 51.6 (CH_2), 49.1 (CH_2), 47.2 (NCH), 44.2 (CH_2), 41.6 ($NHCH_2$), 25.9 (CH), 25.5 (CH); MS: *m*/*z* (%) = 632 [M^+] (1), 434 [M–NBn_2]$^+$ (1), 149 (100); HRMS–EI: *m*/*z* calcd for $C_{29}H_{30}Cl_3N_6O_2S$ [M+H]$^+$: 631.1217.

3,3'-((*Z*)-4-Chloro-2,4-dinitrobuta-1,3-diene-1,1-diyl)bis(*N,N*-dibenzyl-3-azabicyclo-[3.1.0]hexan-6-amine) (**13**)

To a suspension of the dinitrodiene **4** (247 mg, 1.0 mmol) in MeOH (5 mL) was added, with stirring, a solution of the amine **1** (1.14 g, 4.1 mmol) in MeOH (5 mL) at −40 °C, within 10 min. The resulting mixture was stirred at −40 °C for 1 h and at rt for an additional 1 h. The precipitate was isolated, successively washed with H_2O (2 × 20 mL) and cold MeOH (1 × 5 mL), and was finally dried under reduced pressure. Yield: 584 mg (80%); mp 142–143 °C;

IR (KBr) $\tilde{\nu}$: 3444, 3027, 2921, 1589 (NO_2), 1493, 1454, 1354 (NO_2), 1333, 1270, 1188, 1169, 1123, 999, 797, 752, 700 cm^{-1}; ^1H NMR ($CDCl_3$) δ 9.10 (s, 1 H, =CH), 7.39–7.10 (m, 20 H, Ph), 3.95–3.15 (m, 16 H), 1.62–1.30 (m, 6 H); ^{13}C NMR ($CDCl_3$) δ 158.3 (NCN), 138.5 ($C_{quat.}$), 138.3 ($C_{quat.}$), 129.3 (2 CH), 129.1 (6 CH), 128.2 (8 CH), 128.1 (=CH), 127.4 (CH), 127.2 (CH), 127.0 (CH), 126.9 (CH), 116.6 ($C(NO_2)Cl$), 112.0 (CNO_2), 59.8 ($C_{quat.,phenyl}CH_2$), 59.5 ($C_{quat.,phenyl}CH_2$), 59.2 ($C_{quat.,phenyl}CH_2$), 59.1 ($C_{quat.,phenyl}CH_2$), 55.2 (CH_2), 54.3 (CH_2), 53.6 (CH_2), 53.2 (CH_2), 46.7 (NCH), 46.2 (NCH), 30.3 (CH), 26.5 (CH), 25.8 (CH), 23.1 (CH); MS: *m*/*z* (%) = 730 [M^+] (1), 534 $[M–NBn_2]^+$ (2), 111 (100); HRMS–EI: *m*/*z* calcd for $C_{42}H_{44}ClN_6O_4$ $[M+H]^+$: 731.3113.

1,1-Bis(1*H*-1,2,4-triazol-1-yl)-2-nitro-3,4,4-trichlorobuta-1,3-diene (**14**)

Compound **14** was prepared according to a previously published procedure [6] from 10.0 mmol of the nitrodiene **3** and 1*H*-1,2,4-triazole. Yield: 3.08 g (92%), mp 141–143 °C; IR (KBr) $\tilde{\nu}$: 3137, 3125, 3081, 3012, 2874, 1806, 1673, 1579 (NO_2), 1540, 1514, 1445, 1399, 1334 (NO_2), 1298, 1269, 1227, 1156, 1120, 983, 946, 920, 899, 830, 785, 732, 697, 666, 642, 614 cm^{-1}; ^1H NMR ($CDCl_3$) δ 8.28 (s, 1 H), 8.24 (s, 1 H), 8.20 (s, 1 H), 8.16 (s, 1 H); ^{13}C NMR ($CDCl_3$) δ 155.1 ($C_{triazolyl}$-H), 154.6 ($C_{triazolyl}$-H), 145.9 ($C_{triazolyl}$-H), 145.5 ($C_{triazolyl}$-H), 133.0 (CNO_2), 131.6, 131.4, 118.5; MS: *m*/*z* (%) = 335 [M^+] (2), 300 $[M–Cl]^+$ (94), 267 $[M–triazole]^+$ (16), 97 (100).

(*E*)-1-(1*H*-1,2,4-Triazol-1-yl)-1-(4-ethoxyphenylamino)-2-nitro-3,4,4-trichlorobuta-1,3-diene (**15**)

At 0 °C, a suspension of 3.36 g (10.0 mmol) bis(triazolyl) compound **14** in 50 mL of MeOH was charged with a solution of 1.44 g (10.5 mmol) *p*-phenetidine in 5 mL MeOH. The resulting mixture was kept at 0 °C for 1 h and then at rt for 5 h. After cooling to 10 °C, a solution of 5 mL hydrochloric acid in 250 mL of cold water was added under stirring. After 1 h, the precipitate was isolated and washed with HCl (5%, 30 mL), cold water (2 × 50 mL), and Et_2O (30 mL). Drying under reduced pressure afforded the diene **15** (3.36 g, 83%), mp

136–137 °C; IR (KBr) $\tilde{\nu}$: 3112, 2984, 1639, 1580, 1506 (NO_2), 1449, 1338 (NO_2), 1253, 1124, 1046, 993, 957, 879, 825, 656, 606, 581 cm^{-1}; ^1H NMR ($CDCl_3$) δ 11.50 (br s, 1 H, NH), 8.18 (s, 1 H), 8.05 (s, 1 H), 6.93-6.62 (m, 4 H), 3.97 (q, *J* = 7.0 Hz, 2 H, OCH_2), 1.39 (t, *J* = 7.0 Hz, 3 H, Me); ^{13}C NMR ($CDCl_3$) δ 158.5 (CO), 153.2 ($C_{triazolyl}$-H), 146.5 (C-1), 144.9 ($C_{triazolyl}$-H), 128.8 (C-3), 126.9 (CNH), 124.8 (2 CH, C_{aryl}-2,6), 120.7 (C-4), 117.9 (CNO_2), 115.4 (2 CH, C_{aryl}-3,5), 63.8 (CH_2), 14.8 (Me); MS: *m*/*z* (%) = 403 [M^+] (6), 368 $[M–Cl]^+$ (12), 357 $[M–NO_2]^+$ (2), 251 (10), 163 (100); HRMS–EI: *m*/*z* calcd for $C_{14}H_{12}Cl_3N_5O_3$ $[M]^+$: 403.0006.

tert-Butyl 6-(((*E*)-1,1-dichloro-4-((4-ethoxyphenyl)amino)-3-nitro-4-(1*H*-1,2,4-triazol-1-yl)buta-1,3-dien-2-yl)amino)-3-azabicyclo[3.1.0]hexane-3-carboxylate (**16**)

To a suspension of the nitrodiene **15** (202 mg, 0.5 mmol) in MeOH (3 mL) was added, under stirring, a solution of the amine **2** (208 mg, 1.05 mmol) in MeOH (2 mL) at 0 °C within 10 min. The resulting mixture was stirred at 0 °C for 1 h and at rt for 1 d. The precipitate was isolated, washed with H_2O (2 × 10 mL) and finally dried under reduced pressure. Yield: 198 mg (70%); mp 123–125 °C; IR (KBr) $\tilde{\nu}$: 2982, 2942, 2890, 1657 (C=O), 1605, 1588, 1499 (NO_2), 1449, 1411, 1327 (NO_2), 1245, 1172, 1142, 1050, 1021, 986, 925, 851, 808, 771, 675, 560, 444 cm^{-1}; ^1H NMR ($CDCl_3$) δ 8.16 (s, 1 H, CH), 8.06 (s, 1 H, CH), 6.74 (br s, 4 H, Ph), 3.98 (q, *J* = 7.0 Hz, 2 H, OCH_2), 3.62–3.27 (m, 5 H, 2 NCH_2 and NH), 2.12 (br s, 1 H, NHC*H*), 1.53 (br s, 2 CH), 1.43 (s, 9 H, 3 Me), 1.39 (t, *J* = 7.0 Hz, 3 H, Me), the second NH group was not detected; ^1H NMR (acetone-d_6) δ 8.80 (s, 1 H, CH), 8.16 (s, 1 H, CH), 7.08 (d, *J* = 9.0 Hz, 2 H, Ar), 6.88 (d, *J* = 9.0 Hz, 2 H, Ar), 4.03 (q, *J* = 7.0 Hz, 2 H, OCH_2), 3.64 (d, *J* = 11.1 Hz, 2 H, NCH_2), 3.48–3.30 (m, 2 H, NCH_2), 2.45 (br s, 1 H, NHC*H*), 2.11–2.08 (m, 2 CH), 1.43 (s, 9 H, 3 Me), 1.36 (t, *J* = 7.0 Hz, 3 H, Me); no NH detected; ^{13}C NMR (acetone-d_6) δ 156.0 (C=O), 153.7 ($C_{triazolyl}$-H), 152.0 (C-O), 146.2 ($C_{quat.}$), 143.0 ($C_{triazolyl}$-H), 140.0 ($C_{quat.}$), 128.8 (CH$C_{quat.}$), 121.9 (2 CH), 120.3 (CNO_2), 114.4 (2 CH), 101.4 (=CCl_2), 79.0 ($C_{quat.}$), 63.2 (OCH_2), 47.3 (NCH_2), 47.2 (NCH_2), 31.0 (CHNH), 28.3 (3 Me), 21.5 (CH), 20.6 (CH), 14.9

(Me); MS: m/z (%) = 565 [M^+] (18), 520 $[M–OEt]^+$ (26), 464 $[M–Boc]^+$ (6), 96 (100); HRMS (CI) m/z calcd for $C_{24}H_{29}Cl_2N_7O_5Na$ $[M+Na]^+$: 588.1505.

4,5-Dichloro-3-(trichloromethyl)isothiazole (**17**)

Compound **17** was prepared according to the published procedure [7] from 100 mmol of the nitrodiene **3** and sulfur. Yield: 14.9 g (55%); mp 34–35 °C (EtOH); IR (KBr) $\tilde{\nu}$: 1494, 1374, 1349, 1111, 997, 856, 786, 697, 678, 530 cm^{-1}; ^{13}C NMR ($CDCl_3$) δ 159.9 (C-3), 151.8 (C-5), 121.7 (C-4), 90.7 (CCl_3); ^{14}N NMR ($CDCl_3$) δ −93.3 ppm.

4,5-Dichloroisothiazole-3-carboxylic acid (**18**)

Compound **18** was prepared according to the published procedure [8] from 100 mmol of the isothiazole **17** and fuming HNO_3. Yield: 17.8 g (90%); mp 175–176 °C; IR (KBr) $\tilde{\nu}$: 3433, 2909, 1724 (C=O), 1440, 1363, 1337, 1208, 1085, 974, 847, 693, 513, 449, 424 cm^{-1}; ^1H NMR ($CDCl_3$) δ 6.67 (br s, 1 H, OH); ^1H NMR (DMSO-d_6) δ 13.74 (br s, 1 H, OH); ^{13}C NMR (DMSO-d_6) δ 160.5 (C=O), 155.5 (C-3), 150.0 (C-5), 124.4 (C-4); ^{13}C NMR (acetone-d_6) δ 160.2 (C=O), 155.8 (C-3), 150.9 (C-5), 125.8 (C-4).

4,5-Dichloroisothiazole-3-carbonyl chloride (**19**)

A mixture of 5.00 g (25.2 mmol) of the carboxylic acid **18** in 30 mL (413 mmol) of $SOCl_2$ was stirred at rt for 1 h, then under reflux for additional 2 h. After removal of excessive thionyl chloride under reduced pressure, the residue was suspended in 50 mL of anhydrous hexane. The resulting precipitate was collected on a suction filter, washed with anhydrous hexane (2 × 10 mL), and finally dried under reduced pressure. Yield: 5.08 g (93%) of **19**; mp 105–107 °C; IR (KBr) $\tilde{\nu}$: 1809 (C=O), 1740, 1477, 1351, 1136, 1053, 960, 935, 835, 765, 674, 516 cm^{-1}. ^{13}C NMR ($CDCl_3$) δ 153.5, 151.7, 151.4, 127.2 (C-4); MS: m/z (%) = 217 [M^+] (10), 182 $[M–Cl]^+$ (100), 152 $[M–C(O)Cl]^+$ (18).

(6-(Dibenzylamino)-3-azabicyclo[3.1.0]hexan-3-yl) (4,5-dichloroisothiazol-3-yl)-methanone (**20**)

To a suspension of 278 mg (1.0 mmol) of the amine **1** and 207 mg (1.5 mmol) of K_2CO_3 in 2 mL of anhydrous THF was added at rt 217 mg (1.0 mmol) of the isothiazole **19**. The resulting mixture was stirred at rt for 1 d. After removal of the solvent, the residue was treated with cold HCl (1%, 30 mL) for 20 min. The product was extracted with CH_2Cl_2 (2 × 30 mL). The combined organic layers were washed with brine and dried over anhydrous $CaCl_2$. Removal of the solvent under reduced pressure gave the crude product, which was purified by column chromatography on silica gel (petroleum ether/ethyl acetate 5:1). Yield: 293 mg (64%) of **20**; mp 103–105 °C; IR (KBr) $\tilde{\nu}$: 3040, 2997, 2910, 2863, 1637, 1494, 1454, 1350, 1246, 1204, 1126, 1100, 1029, 982, 896, 833, 754, 732, 703 cm^{-1}; ^{1}H NMR ($CDCl_3$) δ 7.33–7.10 (m, 10 H), 3.78 (d, *J* = 12.6 Hz, 1 H, NCH_2), 3.59 (d, *J* = 13.3 Hz, 2 H, $C_{quat.}CH_2$), 3.54 (d, *J* = 13.3 Hz, 2 H, $C_{quat.}CH_2$), 3.54 (d, *J* = 13.3 Hz, 2 H, $C_{quat.}CH_2$), 3.48 (dd, *J* = 11.1, 2.6 Hz, 1 H, NCH_2), 3.37 (dd, *J* = 12.6, 2.6 Hz, 1 H, NCH_2), 3.31 (d, *J* = 11.1 Hz, 1 H, NCH_2), 1.54 (t, *J* = 2.3 Hz, 1 H, NCH), 1.35 (dd, *J* = 4.2, 2.3 Hz, 2 H, CH); ^{13}C NMR ($CDCl_3$) δ 160.5 (C=O), 160.4 (SCCl), 149.1 (C=N), 138.3 (2 $C_{quat.}$), 129.3 (4 CH), 128.1 (4 CH), 127.1 (2 CH), 122.6 (CCl), 59.0 (2 $C_{quat.}CH_2$), 49.7 (NCH_2), 48.0 (NCH_2), 47.3 (NCH), 25.5 (CH), 24.5 (CH); ^{15}N NMR ($CDCl_3$) δ −319.5 (NBn), −244.2 (NCO), −65.8 (C=N); MS: *m*/*z* (%) = 457 [M^+] (1), 366 [M–CH_2Ph]$^+$ (11), 249 (18), 180 [M–*N*,*N*-dibenzyl-3-azabicyclo[3.1.0]hexan-6-amine]$^+$, 91 [CH_2Ph] (100); HRMS–EI: *m*/*z* calcd for $C_{23}H_{22}Cl_2N_3OS$ [M+H]$^+$: 458.0861.

tert-Butyl 6-(4,5-dichloroisothiazole-3-carboxamido)-3-azabicyclo[3.1.0]hexane-3-carboxylate (**21**)

To a suspension of 198 mg (1.0 mmol) of the amine **2** and 207 mg (1.5 mmol) of K_2CO_3 in 2 mL of anhydrous THF was added at rt 217 mg (1.0 mmol) of the isothiazole **19**. The resulting mixture was stirred at rt for 1 d. After removal of the solvent, the residue was treated with cold HCl (1%, 30 mL). The resulting precipitate was filtered off and successively

washed with water (2 × 5 mL), NaOH (5%, 2 × 5 mL), water (2 × 5 mL), and cold Et_2O (1 × 3 mL). Drying under reduced pressure gave the isothiazole **21** (227 mg, 60%); mp 136–137 °C; IR (KBr) $\tilde{\nu}$: 3388, 3302, 1696, 1669 (C=O), 1534, 1479, 1402, 1244, 1181, 1119, 1005, 961, 922, 874, 851, 782, 734, 677, 628, 555, 514, 446 cm^{-1}; ^1H NMR ($CDCl_3$) δ 7.22 (br s, 1 H, NH), 3.75 (dd, *J* = 10.9, 2.8 Hz, 2 H, NCH_2), 3.42 (dd, *J* = 10.9, 2.8 Hz, 2 H, NCH_2), 2.58 (br s, 1 H, NCH), 1.81 (br s, 2 H, CH), 1.44 (s, 9 H, Me); ^{13}C NMR ($CDCl_3$) δ 159.8 (C(O)NH), 156.2 (C(O)O), 154.4 (C=N), 150.6 (SC), 124.9 (CCl), 79.6 (*C*Me), 47.6 (NCH_2), 47.4 (NCH_2), 32.3 (NHCH), 28.4 (3 C, Me), 25.0 (CH), 23.7 (CH); MS: *m*/*z* (%) = 377 [M^+] (2), 276 $[M–Boc]^+$ (60), 180 [M–*tert*-butyl aminoazabicyclohexane-3-carboxylate]$^+$ (100); HRMS–EI: *m*/*z* calcd for $C_{14}H_{17}Cl_2N_3O_3S$ $[M]^+$: 377.0368.

1,1-Bis(benzotriazol-1-yl)-2-nitro-3,4,4-trichlorobuta-1,3-diene (**22**)

Compound **22** was prepared according to the published literature [9] from the nitrodiene **3** and 1*H*-benzotriazole. Yield: 76%. All spectral data were in accordance with the literature.

2-(1*H*-Benzotriazol-1-yl)-4-(dichloromethylene)-3-nitro-4*H*-pyrido[1,2-*a*]pyrimidine (**23**)

To a solution of 4.37g (10.0 mmol) of the nitrodiene **22** in 30 mL of THF was added at rt 2.82 g (30.0 mmol) of 2-aminopyridine within 5 min. The resulting mixture was stirred for 8 h. Subsequently, the solvent was removed under reduced pressure. The residue was treated with cold hydrochloric acid (10%, 60 mL) for 1 h. The precipitate was filtered off, washed with water (30 mL) and cold MeOH (20 mL), and then dried under reduced pressure. Yield: 3.15 g (84%) of the pyrimidine **23**; mp 158–160 °C; IR (KBr) $\tilde{\nu}$: 3082, 1628, 1608, 1552, 1507, 1469, 1434, 1360, 1307, 1215, 1131, 1047, 992, 959, 887, 822, 755, 737, 688, 654, cm^{-1}; ^1H NMR (DMSO-d_6) δ 8.99 (dd, *J* = 6.8, 0.9 Hz, 1 H, C_6-H), 8.32-8.22 (m, 1 H), 8.23 (d, *J* = 7.8 Hz, 1 H), 7.92 (d, *J* = 8.3 Hz, 1 H), 7.86 (d, *J* = 8.3 Hz, 1 H, C_9-H), 7.70 (dd, *J* = 8.3, 7.8 Hz, 1 H), 7.64-7.50 (m, 2 H); ^{13}C NMR (DMSO-d_6) δ 151.3 (C-9a), 148.0 (C-2), 145.6, 143.6 (CH), 138.8 (CH), 132.3, 129.6 (CH), 126.3, 125.5 (CH), 123.7 (CH), 121.3 (CCl_2 or CNO_2), 120.0 (CH), 119.2 (CH), 113.0 (CH), 102.3 (CNO_2 or CCl_2); MS: *m*/*z* (%) = 374 [M^+]

(1), 256 [M–benzotriazole]$^+$ (14), 219 (18), 119 [benzotriazole] (14), 92 (100); HRMS–EI: *m*/*z* calcd for $C_{15}H_9Cl_2N_6O_2$ [M+H]$^+$: 375.0164.

N,N-Dibenzyl-3-(4-(dichloromethylene)-3-nitro-4*H*-pyrido[1,2-*a*]pyrimidin-2-yl)-3-azabicyclo[3.1.0]hexan-6-amine (**24**)

To a solution of 375 mg (1.0 mmol) of the pyrimidine **23** in 5 mL of MeOH was added at 0 °C 585 mg (2.1 mmol) of the amine **1** within 5 min. After 1 h at 0 °C and an additional 5 h at rt, the precipitate was filtered off, washed with cold HCl (5%, 3 mL) and water (5 mL), then dried under reduced pressure. Yield: 460 mg (86%) of the pyrimidine **24**; mp 78–79 °C; IR (KBr) $\tilde{\nu}$: 3424, 3026, 2877, 1638, 1547, 1494, 1454, 1368, 1326, 1255, 1149,1074, 935, 888, 751, 700 cm^{-1}; ^{1}H NMR ($CDCl_3$) δ 8.10 (d, *J* = 7.0 Hz, 1 H), 7.64-7.56 (m, 1 H), 7.36–7.22 (m, 10 H), 7.12 (d, *J* = 8.0 Hz, 1 H), 6.83-6.74 (m, 1 H), 3.66 (br s, 4 H), 3.63 (br s, 2 H), 3.16 (br s, 2 H), 1.43 (br s, 1 H), 1.38 (br s, 2 H); ^{13}C NMR ($CDCl_3$) δ 155.3, 151.3, 139.4 (CH), 138.6, 138.1, 135.7 (CH), 129.3 (2 CH), 129.2 (2 CH), 128.2 (4 CH), 127.1 (2 CH), 125.4, 123.3 (CH), 115.1 (CH), 112.7 (CNO_2), 95.4 (CCl_2), 59.4 ($C_{quat.}CH_2$), 58.7 ($C_{quat.}CH_2$), 49.2 (NCH_2), 47.2 (NCH_2), 45.2 (NCH), 25.4 (CH), 23.7 (CH); MS: *m*/*z* (%) = 533 [M$^+$] (2), 277 [*N*,*N*-dibenzyl-3-azabicyclo[3.1.0]hexan-6-amine] (7), 256 [M–*N*,*N*-dibenzyl-3-azabicyclo[3.1.0]hexan-6-amine]$^+$ (3), 205 (12), 158 (32), 119 (100); HRMS–EI: *m*/*z* calcd for $C_{28}H_{26}Cl_2N_5O_2$ [M+H]$^+$: 534.1464.

N,N-Dibenzyl-3-(1-(benzotriazol-1-yl)-2-nitro-3,4,4-trichlorobuta-1,3-dienyl)-3-azabicyclo[3.1.0]hexan-6-amine (**25**)

To a suspension of 437 mg (1.0 mmol) of the nitrodiene **22** in 5 mL of MeOH was added dropwise a solution of 292 mg (1.05 mmol) of the amine **1** in 3 mL of MeOH at 0 °C within 2 min. The resulting mixture was stirred at 0 °C for 1 h and at rt for 3 h. Thereafter, 1 mL of conc. HCl was added and the mixture was stirred for an additional 20 min. The formed precipitate was collected on a suction filter, washed with water (2 × 10 mL) and cold MeOH (1 × 5 mL), and then finally dried under reduced pressure. Yield: 566 mg (95%); mp 189–191

°C; IR (KBr) $\tilde{\nu}$: 3426, 3065, 1560 (NO_2), 1503, 1454, 1286 (NO_2), 1226, 1030, 911, 862, 816, 783, 748, 701 cm^{-1}; ^1H NMR ($CDCl_3$) δ 8.20 (d, J = 8.2 Hz, 1 H), 7.92-7.30 (m, 13 H), 4.49 (br s, 2 H), 4.24 (br s, 2 H), 3.70-2.90 (m, 4 H), 2.53 (br s, 1 H), 2.18 (br s, 2 H); ^{13}C NMR ($CDCl_3$) δ 146.0, 145.7, 138.5 (2 $C_{quat.}$), 132.0, 131.8 (4 CH), 130.2 (CH), 128.0, 129.2 (4 CH), 126.2 (2 CH), 126.0 (CH), 123.6, 121.0 (CH), 116.2 (CNO_2), 109.9 (CH), 60.0 (2C, $C_{quat.}CH_2$), 52.9 (NCH_2), 50.3 (NCH_2), 45.0 (NCH), 21.2 (CH), 20.8 (CH); MS: *m*/*z* (%) = 594 [M^+] (1), 503 [$M–PhCH_2]^+$ (20), 441 [M–Cl-benzotriazole]$^+$ (12), 277 [*N*,*N*-dibenzyl-3-azabicyclo[3.1.0]hexan-6-amine] (22), 236 (90), 158 (100); HRMS–EI: *m*/*z* calcd for $C_{29}H_{26}Cl_3N_6O_2$ $[M+H]^+$: 595.1183.

N,N-Dibenzyl-3-(6-(dichloromethyl)-2-methyl-5-nitropyrimidin-4-yl)-3-azabicyclo-[3.1.0]hexan-6-amine (**26**)

To a suspension of 71 mg (2.96 mmol) of sodium hydride (118 mg of 60% NaH in mineral oil washed twice with anhydrous pentane) in 10 mL of anhydrous THF was added 210 mg (2.22 mmol) of acetamidine hydrochloride. After this slurry was cooled down to −10 °C a solution of 442 mg (0.741 mmol) of the butadiene **25** in 3 mL of THF was added dropwise. The resulting mixture was stirred at −10 °C for 1 h, and then was kept at rt overnight. Subsequently, the reaction mixture was poured into a solution of 3 mL of conc. hydrochloric acid in 30 mL of cold water. After 10 min of stirring, the crude product was extracted with CH_2Cl_2 (3 × 40 mL). The combined organic phases were washed with water (2 × 20 mL), the solvent was distilled off and the residue dried in vacuo. After column chromatography on silica gel (petroleum ether/ethyl acetate 5:1) the pyrimidine **26** was obtained in 58% yield (289 mg); mp 144–146 °C; IR (KBr) $\tilde{\nu}$: 3386, 2925, 2790, 2598, 1574 (NO_2), 1537, 1457, 1427, 1340 (NO_2), 1213, 1032, 878, 745, 701 cm^{-1}; ^1H NMR ($CDCl_3$) δ 7.36–7.31 (m, 4 H), 7.30–7.23 (m, 6 H), 6.78 (s, 1 H, $CHCl_2$), 3.98–3.93 (m, 2 H, NCH_2), 3.65 (br s, 4 H, $C_{quat.}CH_2$), 3.44 (d, J = 10.0 Hz, 2 H, NCH_2), 2.60 (s, 3 H, Me), 1.86–1.78 (m, 2 H, CH), 1.47 (br s, 1 H, NCH); ^{13}C NMR ($CDCl_3$) δ 168.3 (*C*Me), 155.2, 151.5, 138.1 (2 $C_{quat.}$), 129.3 (4 CH), 128.2 (4 CH), 127.2 (2 CH), 119.9 (CNO_2), 65.3 ($CHCl_2$), 59.0 (2 $C_{quat.}CH_2$), 50.0

(NCH_2), 49.9 (NCH_2), 48.1 (NCH), 26.0 (Me), 23.9 (CH), 23.8 (CH); MS: *m*/*z* (%) = 497 [M^+] (1), 406 [M–*N*,*N*-dibenzyl-3-azabicyclo[3.1.0]hexan-6-amine]$^+$ (2), 289 (4), 196 [NBn_2] (5), 91 [Bn] (100); HRMS–EI: *m*/*z* calcd for $C_{25}H_{26}Cl_2N_5O_2$ [M+H]$^+$: 498.1464.

N,N-Dibenzyl-3-(1-(4-chlorophenyl)-5-(dichloromethyl)-4-nitro-1*H*-pyrazol-3-yl)-3-aza-bicyclo[3.1.0]hexan-6-amine (**27**)

To a suspension of 298 mg (0.5 mmol) of the benzotriazole **25** and 197 mg (1.1 mmol) of 4-chlorophenylhydrazine hydrochloride in 5 mL of MeOH was added 68 mg (1.25 mmol) of sodium methoxide at 0 °C within 5 min. After 1 h at 0 °C and 1 d at rt, the resulting precipitate was filtered off and washed successively with cold HCl (5%, 2 × 5 mL), water (1 × 5 mL), and cold MeOH (1 × 3 mL). Drying under reduced pressure afforded 218 mg (75%) of the pyrazole **27**; mp 131–132 °C; IR (KBr) $\tilde{\nu}$: 3449, 3037, 2910, 2849, 1572 (NO_2), 1492, 1398, 1364, 1337 (NO_2), 1273, 1192, 1092, 1015, 958, 833, 755, 702, 644, 523 cm^{-1}; ^1H NMR ($CDCl_3$) δ 7.52-7.46 (br s, 4 H), 7.36 (s, 1 H, $CHCl_2$), 7.35-7.20 (m, 10 H), 3.67 (br s, 4 H, $C_{quat.}CH_2$), 3.59 (d, *J* = 10.2 Hz, 2 H, NCH_2), 3.26 (d, *J* = 10.2 Hz, 2 H, NCH_2), 1.76 (br s, 1 H, NCH), 1.46 (br s, 2 H, CH); ^{13}C NMR ($CDCl_3$) δ 150.1 (C=N), 138.4 (2 $C_{quat.}$), 137.8, 137.0, 136.3, 129.5 (4 CH), 128.1 (8 CH), 127.0 (2 CH), 121.2 (CNO_2), 59.0 (2 $C_{quat.}CH_2$), 58.0 ($CHCl_2$), 50.9 (2 NCH_2), 46.6 (NCH), 25.5 (2 CH); MS: *m*/*z* (%) = 581 [M^+] (3), 565 [M–O]$^+$ (3), 546 [M–Cl]$^+$ (3), 510 (22), 490 [M–Bn]$^+$ (16), 373 (76), 158 (100); HRMS–EI: *m*/*z* calcd for $C_{29}H_{27}Cl_3N_5O_2$ [M+H]$^+$: 582.1230.

N,N-Dibenzyl-3-(1-(2,6-dichloro-4-(trifluoromethyl)phenyl)-5-(dichloromethyl)-4-nitro-1*H*-pyrazol-3-yl)-3-azabicyclo[3.1.0]hexan-6-amine (**28**)

To a suspension of 298 mg (0.5 mmol) of the benzotriazole derivative **25** in 5 mL of MeOH was added at rt 319 mg (1.3 mmol) of 2,6-dichloro-4-(trifluoromethyl)phenylhydrazine. The reaction mixture was heated with stirring under reflux for 5 d. Then, the mixture was concentrated to a volume of about 2 mL on a rotary evaporator. Addition of cold hydrochloric acid (5%, 20 mL) led to a precipitate, which was filtered off, washed with cold HCl (5%, 20 mL) and cold water (5 mL). The product was dried under reduced pressure. Yield: 308 mg

(90%) of pyrazole **28**; mp 127–128 °C; IR (KBr) $\tilde{\nu}$: 3425, 3036, 2878, 1571 (NO_2), 1499, 1395, 1321 (NO_2), 1210, 1144, 1104, 1031, 953, 882, 837, 819, 747, 702 cm^{-1}; ^{1}H NMR ($CDCl_3$) δ 7.76 (br s, 2 H, Ar), 7.66–7.56 (m, 4 H), 7.54–7.42 (m, 6 H), 7.30 (s, 1 H, $CHCl_2$), 4.51 (d, J = 12.4 Hz, 2 H, $C_{quat.}CH_2$), 4.15 (d, J = 12.4 Hz, 2 H, $C_{quat.}CH_2$), 3.50 (d, J = 10.3 Hz, 2 H, NCH_2), 3.10 (d, J = 10.3 Hz, 2 H, NCH_2), 2.31–2.18 (m, 3 H, CH); ^{13}C NMR ($CDCl_3$) δ 150.7 (C=N), 139.7, 136.8, 136.6 (2 $C_{quat.}$), 134.7 ($C$$CF_3$, $^2J_{C,F}$ = 34.5 Hz), 131.9 (4 CH), 130.2 (2 CH), 129.3 (4 CH), 128.2 (2 CCl), 126.0 (2 CH, $^3J_{C,F}$ = 4.3 Hz), 121.8 (CF_3, $^1J_{C,F}$ = 278.0 Hz), 121.7 (CNO_2), 59.3 (2 $C_{quat.}CH_2$), 57.4 ($CHCl_2$), 49.7 (2 NCH_2), 42.0 (NCH), 22.6 (2 CH); MS: m/z (%) = 683 [M^+] (3), 667 $[M–O]^+$ (2), 648 $[M–Cl]^+$ (4), 612 (14), 592 $[M–Bn]^+$, 487 $[M–NBn_2]^+$, 477 (60), 158 (100); HRMS–EI: m/z calcd for $C_{30}H_{25}Cl_4N_5O_2F_3$ $[M+H]^+$: 684.0714.

N-(3-Azabicyclo[3.1.0]hexan-6-yl)-4,5-dichloroisothiazole-3-carboxamide (**29**)

50.0 mg (0.13 mmol) of isothiazole **21** was added at 0 °C within 5 min to 2 mL of trifluoroacetic acid. The resulting mixture was stirred at 0 °C for 1 h. After warming up to rt the mixture was kept at this temperature for an additional 3 h. Excessive trifluoroacetic acid was removed in vacuo. The residue was treated for 30 min with 30 mL of cold water and 300 mg of $NaHCO_3$ and was then extracted with chloroform (3 × 30 mL). The combined organic layers were washed with water (1 × 10 mL) and dried over calcium chloride. After removal of the solvent and drying in vacuo the isothiazole **29** was obtained as a bright yellow solid (30.5 mg, 83%); mp 190–195 °C; IR (KBr) $\tilde{\nu}$: 3329, 3272, 3004, 2925, 2863, 1659 (C=O), 1525, 1417, 1350, 1260, 848, 750, 737 cm^{-1}; ^{1}H NMR ($CDCl_3$) δ 7.20 (br s, 1 H, NHCO), 3.17 (d, J = 11.5 Hz, 2 H, CH_2), 2.92 (d, J = 11.5, 2 H, CH_2), 2.58 (dd, J = 5.0, 2.4 Hz, 1 H, NCH), 1.73 (br s, 1H, NH), 1.67 (br s, 2 H, CH); ^{13}C NMR ($CDCl_3$) δ 159.7 (C=O), 156.4 (C=N), 150.4 (SC), 124.8 (CCl), 48.5 (CH_2), 29.4 (NHCH), 25.7 (2 CH); MS: m/z (%) = 277 [M^+] (1), 242 $[M–Cl]^+$ (7), 180 $[M–NH(C_5H_7NH)]^+$ (100), 152 $[M–CONH(C_5H_7NH)]^+$ (23); HRMS–EI: m/z calcd for $C_9H_{10}Cl_2N_3OS$ $[M+H]^+$: 277.9922.

(E)-1-(1-((2-Chlorothiazol-5-yl)methyl)imidazolidin-2-ylidene)-1-nitropropan-2-one (**30**)

A solution of 90.0 mg (0.14 mmol) of the imidazolidine **12** in anhydrous EtOH (20 mL) was hydrogenated with hydrogen at atmospheric pressure by shaking for 3 d. After filtration over celite and evaporation of the solvent, the residue was purified by column chromatography (eluent: PE/EE 2:1). The nitropropanone **30** was obtained as a yellow solid (19 mg, 44%); mp 154–155 °C; IR (KBr) $\tilde{\nu}$: 3267, 2925, 1606 (C=O), 1564 (NO_2), 1526, 1440, 1349 (NO_2), 1301, 1244, 1154, 1051, 981, 927, 678, 597 cm^{-1}; ^1H NMR ($CDCl_3$) δ 9.03 (br s, 1H, NH), 7.49 (s, 1H, $N_{thiazolyl}CH$), 4.52 (br s, 2 H, $C_{thiazolyl}CH_2$), 3.93–3.86 (m, 2 H), 3.76-3.70 (m, 2 H), 2.57 (s, 3 H, Me); ^{13}C NMR ($CDCl_3$) δ 191.2 (C=O), 163.2 (NCN), 154.6 (NCCl), 141.7 ($C_{thiazolyl}H$), 133.1 ($C_{quat.,thiazolyl}$), 115.4 (CNO_2), 48.3 (CH_2), 45.6 (CH_2), 42.4 (CH_2), 31.0 (Me); MS: m/z (%) = 302 [M^+] (4), 287 $[M–Me]^+$ (1), 256 $[M–NO_2]^+$ (27), 220 $[M–NO_2–HCl]^+$ (28), 132 (100); HRMS–EI: m/z calcd for $C_{10}H_{10}ClN_4O_3S$ $[M–H]^+$: 301.0162.

(N)-Benzyl-3-(1-(2,6-dichloro-4-(trifluoromethyl)phenyl)-5-methyl-4-nitro-1H-pyrazol-3-yl)-3-azabicyclo[3.1.0]hexan-6-amine (**31**)

Compound **31** was prepared from 100.0 mg (0.146 mmol) of the pyrazole **28** by following a procedure analogous to that described for **30**. The mixture was shaken for 1 d. Yield: 35.0 mg (45%) of pyrazole **30**; mp 128–130 °C; IR (KBr) $\tilde{\nu}$: 3418, 2931, 2856, 1570 (NO_2), 1487, 1395, 1365, 1322 (NO_2), 1210, 1177, 1142, 1111, 882, 831, 819, 752, 701 cm^{-1}; ^1H NMR ($CDCl_3$) δ 9.74 (br s, 1 H, NH), 7.74 (br s, 2 H, Ar), 7.61 (d, J = 7.2 Hz, 2 H), 7.43 (t, J = 7.2 Hz, 2 H), 7.36 (t, J = 7.2 Hz, 1 H), 4.14 (br s, 2 H, NHCH_2), 3.77 (d, J = 10.0 Hz, 2 H, NCH_2), 3.23 (d, J = 10.0 Hz, 2 H, NCH_2), 2.47 (br s, 1 H, NHCH), 2.36 (s, 3 H, Me), 2.22 (br s, 2 H, CH); ^{13}C NMR ($CDCl_3$) δ 151.27 (C=N), 143.8, 136.4, 136.2 (2 CCl), 134.1 ($C$$CF_3$, ^2J (C,F) = 34.5 Hz), 130.8 (2 CH), 130.1 (C-Me), 129.5 (1 CH), 129.1 (2 CH), 126.0 (2 CH, ^3J (C,F) = 3.8 Hz), 124.0 (CNO_2), 121.0 (CF_3, ^1J (C,F) = 273.6 Hz), 52.0 ($C_{quat.}CH_2$), 49.9 (2 NCH_2), 36.1 (NHCH), 21.3 (2 CH), 12.4 (Me); MS: m/z (%) = 526 [M^+] (1), 419 $[M–NHBn]^+$ (1), 338 $[M–N(C_5H_7)NHBn]^+$ (2), 213 (Cl_2CF_3Ph) (5), 91 (100); HRMS–EI: m/z calcd for $C_{23}H_{21}Cl_2N_5O_2F_3$ $[M+H]^+$: 526.1024.

References

1. de Meijere, A.; Williams, C. M.; Kourdioukov, A.; Sviridov, S. V.; Chaplinski, V.; Kordes, M.; Savchenko, A. I.; Stratmann, C.; Noltemeyer, M. *Chem.–Eur. J.* **2002,** *8,* 3789–3801. doi:10.1002/1521-3765(20020816)8:16<3789::AID-CHEM3789>3.0.CO;2-R
2. Potkin, V. I.; Zapol'skii, V. A.; Kaberdin, R. V. *Izv. Akad. Nauk Bel., Ser. Khim. Nauk* **1996,** *40,* 68–71.
3. Zapol'skii, V. A.; Potkin, V. I.; Kaberdin, R. V. *Russ. J. Org. Chem.* **1994,** *30,* 1435–1445.
4. Zapol'skii, V. A.; Nutz, E.; Namyslo, J. C.; Adam, A. E. W.; Kaufmann, D. E. *Synthesis* **2006,** 2927–2933. doi:10.1055/s-2006-950187
5. Zapol'skii, V. A.; Fischer, R.; Namyslo, J. C.; Kaufmann, D. E. *Bioorg. Med. Chem.* **2009,** *17,* 4206–4215. doi:10.1016/j.bmc.2009.01.001
6. Zapol'skii, V. A.; Potkin, V. I.; Nechai, N. I.; Kaberdin, R. V.; Pevzner, M. S. *Russ. J. Org. Chem.* **1997**, *33*, 1632–1637.
7. Kaberdin, R. V.; Potkin, V. I.; Ol'dekop, Y. A. *Dokl. Chem.* **1988**, *300*, 173–175.
8. Kaberdin, R. V.; Potkin, V. I.; Ol'dekop, Y. A. *Russ. J. Org. Chem.* **1990,** *26,* 1347–1351.
9. Zapol'skii, V. A.; Potkin, V. I.; Nechai, N. I.; Kaberdin, R. V. *Russ. J. Org. Chem.* **1993,** *29,* 5, 731–734.
